# Associations Between Social Cognitive Determinants and Movement-Related Behaviors in Studies Using Ecological Momentary Assessment Methods: Systematic Review

**DOI:** 10.2196/44104

**Published:** 2023-04-07

**Authors:** Kelsey M Bittel, Kate Y O'Briant, Rena M Ragaglia, Lake Buseth, Courtney Murtha, Jessica Yu, Jennifer M Stanely, Brynn L Hudgins, Derek J Hevel, Jaclyn P Maher

**Affiliations:** 1 Department of Kinesiology University Of North Carolina Greensboro Greensboro, NC United States

**Keywords:** motivation, psychosocial, physical activity, sedentary behavior, ambulatory assessment, mobile phone

## Abstract

**Background:**

The social cognitive framework is a long-standing framework within physical activity promotion literature to explain and predict movement-related behaviors. However, applications of the social cognitive framework to explain and predict movement-related behaviors have typically examined the relationships between determinants and behavior across macrotimescales (eg, weeks and months). There is more recent evidence suggesting that movement-related behaviors and their social cognitive determinants (eg, self-efficacy and intentions) change across microtimescales (eg, hours and days). Therefore, efforts have been devoted to examining the relationship between social cognitive determinants and movement-related behaviors across microtimescales. Ecological momentary assessment (EMA) is a growing methodology that can capture movement-related behaviors and social cognitive determinants as they change across microtimescales.

**Objective:**

The objective of this systematic review was to summarize evidence from EMA studies examining associations between social cognitive determinants and movement-related behaviors (ie, physical activity and sedentary behavior).

**Methods:**

Studies were included if they quantitatively tested such an association at the momentary or day level and excluded if they were an active intervention. Using keyword searches, articles were identified across the PubMed, SPORTDiscus, and PsycINFO databases. Articles were first assessed through abstract and title screening followed by full-text review. Each article was screened independently by 2 reviewers. For eligible articles, data regarding study design, associations between social cognitive determinants and movement-related behaviors, and study quality (ie, Methodological Quality Questionnaire and Checklist for Reporting Ecological Momentary Assessment Studies) were extracted. At least 4 articles were required to draw a conclusion regarding the overall associations between a social cognitive determinant and movement-related behavior. For the social cognitive determinants in which a conclusion regarding an overall association could be drawn, 60% of the articles needed to document a similar association (ie, positive, negative, or null) to conclude that the association existed in a particular direction.

**Results:**

A total of 24 articles including 1891 participants were eligible for the review. At the day level, intentions and self-efficacy were positively associated with physical activity. No other associations could be determined because of conflicting findings or the small number of studies investigating associations.

**Conclusions:**

Future research would benefit from validating EMA assessments of social cognitive determinants and systematically investigating associations across different operationalizations of key constructs. Despite the only recent emergence of EMA to understand social cognitive determinants of movement-related behaviors, the findings indicate that daily intentions and self-efficacy play an important role in regulating physical activity in everyday life.

**Trial Registration:**

PROSPERO CRD42022328500; https://www.crd.york.ac.uk/prospero/display_record.php?RecordID=328500

## Introduction

### Background

The World Health Organization recommends that adults engage in at least 150 minutes of moderate-intensity or 75 minutes of vigorous-intensity physical activity (PA; or an equivalent combination of both) per week and limit the amount of time spent engaging in sedentary behavior (SB) [1]. Despite these recommendations, approximately 28% of adults do not meet PA guidelines [2]. Furthermore, on average, adults engage in 8.2 hours per day of SB [3]. This represents a considerable health burden as individuals who are physically inactive and do not meet PA guidelines have a 20% to 30% increased chance of premature death versus individuals who are active and meet PA guidelines [2,4]. Physical inactivity and SB can also lead to chronic diseases and are a contributing factor to 35 pathological and clinical conditions (eg, obesity, type 2 diabetes, and cardiovascular diseases) [5]. Considering the risks associated with physical inactivity and SB, understanding the factors that influence movement-related behaviors such as PA and SB is paramount.

There are a number of different theoretical approaches to understand and explain PA and SB engagement (eg, the humanistic framework, the dual process framework, and ecological models) [6]. Social cognitive framework represents one of the most used and long-standing theoretical frameworks within the movement-related behavior literature to explain and predict behavioral engagement [6]. Social cognitive framework emphasizes key determinants of behavior as individuals’ cognitions about the anticipated outcomes of a behavior and their ability to engage in a behavior [7,8]. In line with these theories, individuals will focus their efforts toward (eg, intentions) and subsequently engage in a behavior if their beliefs about the behavior are positive (eg, outcome expectations) and they are confident in their ability to engage in the behavior (eg, self-efficacy) [9-13]. There are 2 prominent theories that are considered part of the social cognitive framework are Social Cognitive Theory and the Theory of Planned Behavior [9,10].

Specifically, Social Cognitive Theory identifies self-efficacy—​an individual’s belief in their ability to engage in a behavior—and outcome expectations—the perception of consequences (positive or negative) of an individual’s action—as key determinants of behavior [8,10]. The Theory of Planned Behavior posits that intention, or one’s willingness to try to engage in a behavior, is the main antecedent of behavior [7,9]. The Theory of Planned Behavior also proposes that intention formation is predicted by three factors: (1) attitudes or the extent to which one has positive or negative evaluations of a behavior, (2) subjective norms or the perceived social pressure to engage in a behavior, and (3) perceived behavioral control or the extent to which a person has the capacity and free will to engage in a behavior [7,9]. Furthermore, both theories acknowledge that facilitators and barriers can help or hinder engagement in a behavior, respectively [7-10]. Therefore, these theories outline social cognitive determinants hypothesized to influence movement-related behaviors.

Several reviews have indicated that social cognitive determinants predict PA behavior in observational studies. However, there is experimental evidence indicating that the extent to which these determinants predict changes in behavior is generally much weaker [14-16]. Within the last decade, evidence has accumulated suggesting that PA and SB are independent health behaviors [17,18], and studies have applied social cognitive frameworks to explain and predict SB. Findings across SB studies appear to mirror those of PA studies, with evidence for relationships between social cognitive determinants and behavior indicating stronger relationships in observational studies than in experimental studies [19-21].

A potential reason for the limited effectiveness of social cognitive determinants in explaining and predicting PA and SB is that the timescale in which these relationships are assessed is not the timescale in which social cognitive determinants influence decisions to engage in a behavior [10,22]. Most of the research investigating associations between social cognitive determinants and PA or SB tends to assess these constructs infrequently and ask participants to report their usual level of social cognitive determinants or behavior over macrotimescales (eg, weeks and months) [21,23]. However, PA and SB are repeat-occurrence behaviors, meaning that these behaviors are typically engaged in multiple times per week or even multiple times per day [24]. Furthermore, the day is an elemental structure in human life. Days are easily defined and universally experienced because of the light-dark cycles of the sun and associated sleep-wake cycles. Moreover, people self-regulate and restore self-regulatory resources throughout these cycles [25]. The changing contexts of people’s daily lives could also influence daily and within-day motivation and movement-related behaviors. Therefore, methods that capture typical levels of movement-related behaviors and social cognitive determinants on a macrotimescale may overlook important information regarding decisions to engage in a bout of PA or SB across microtimescales (eg, hours and days). Previous research has documented that social cognitive determinants, PA, and SB are dynamic, varying within individuals across time and space [26-28]. Investigating associations between social cognitive determinants and movement-related behaviors in the content of daily life across microtimescales can elucidate the motivational determinants of movement-related behaviors and potentially enhance intervention efforts.

Recent evidence of the dynamic and time-varying nature of PA and SB has increased in part from advances in methodology to assess behavior and its determinants. Ecological momentary assessment (EMA) has gained popularity over the last decade in the movement-related behavior literature as a methodology to capture fluctuations in behavior and social cognitive determinants across microtimescales in naturalistic settings [29,30]. EMA is a real-time data capture methodology that repeatedly assesses individuals on a phenomenon of interest in their natural environment (eg, motivation and behavior) [31]. EMA is useful for assessing phenomena that change across time and space, such as movement-related behaviors and their determinants. For instance, an individual’s engagement in PA behaviors may change over the course of the day, as may their feelings of confidence (ie, self-efficacy) in engaging in PA. To study these fluctuations in behavior as well as how these 2 constructs might covary, a smartphone-based EMA protocol could assess self-efficacy at predetermined (eg, every 2 hours) or randomly occurring (eg, anytime between 8 AM and 8 PM) times throughout the day. In addition, monitoring via an accelerometer could be conducted. In an EMA protocol, when participants receive a notification to complete a questionnaire through an app or website on their smartphone that measures self-efficacy to engage in PA over the following 2 hours, they are asked to briefly stop what they are doing to complete the questionnaire. Responses on the smartphone are date- and time-stamped to facilitate easy pairing of the smartphone questionnaire and accelerometer data in the 2-hour window (referenced in the self-efficacy assessment) after the EMA prompt. As these notifications happen repeatedly, researchers are able to capture these constructs and their associations as individuals go about their day-to-day activities, allowing them to examine these associations across the changing contexts of everyday life.

Therefore, EMA methodology can reduce recall biases and enhance ecological validity by evaluating a phenomenon of interest close in time to when it occurs in real-world settings as individuals go about their normal day-to-day lives [29]. Today, EMA protocols can be delivered through various media (ie, apps on mobile devices, internet-based questionnaires, and SMS text messages) that can provide time stamps of participant responses. As noted, this can facilitate the pairing of EMA responses with other time-stamped data sources such as accelerometers and allows for the investigation of the temporal sequence of relationships between key constructs. Previous research has established the feasibility and validity of using smartphone-based EMA to assess PA and SB as well as their determinants in diverse populations across their life span [28,32-35].

### Objectives

The use of EMA to capture and understand PA and SB has increased over the past decade. As a result, recent reviews have summarized EMA findings regarding various determinants of PA and SB, including affective states [36] and environmental contexts [37]. However, there is yet to be a review summarizing the associations between social cognitive determinants and movement-related behaviors from studies using EMA methodologies. This systematic review aimed to summarize the literature regarding within-day and day-level associations between social cognitive determinants and movement-related behaviors using within-day or daily EMA methodologies. The decision to focus exclusively on within-day and day-level associations was based on the repeat-occurrence nature of movement-related behaviors and the fact that assessment schedules occurring less frequently than the day level may not be sensitive to the changing contexts in everyday life and the factors driving decisions to engage in occasions of PA or SB. This rationale is bolstered by the fact that the day represents a natural and fundamental reoccurring event in human life.

## Methods

This systematic review was conducted and reported in accordance with the PRISMA (Preferred Reporting Items for Systematic Reviews and Meta-Analyses) guidelines [38] and was registered in PROSPERO (CRD42022328500).

### Inclusion and Exclusion Criteria

Articles that met the following criteria were included in the review: (1) human-participant research, (2) available in English, (3) quantitative data available for at least one association between a social cognitive determinant (ie, the independent variable) and movement-related behavior (ie, the dependent variable), and (4) within-day or daily EMA study design. Social cognitive determinants were defined as constructs specified within Social Cognitive Theory and the Theory of Planned Behavior, 2 popular social cognitive frameworks in the movement-related behavior literature [7-9,22,39]. Therefore, social cognitive determinants of interest for this systematic review included intentions, attitudes, subjective norms, perceived behavioral control, self-efficacy, outcome expectations, risk perceptions, barriers, facilitators, goals, and plans regarding movement-related behaviors. To focus on naturally occurring associations between social cognitive determinants and movement-related behaviors, articles were excluded if they used an active experimental design. In addition, articles were excluded if they were not published in a peer-reviewed scholarly journal.

### Search Process

**Figure 1:**
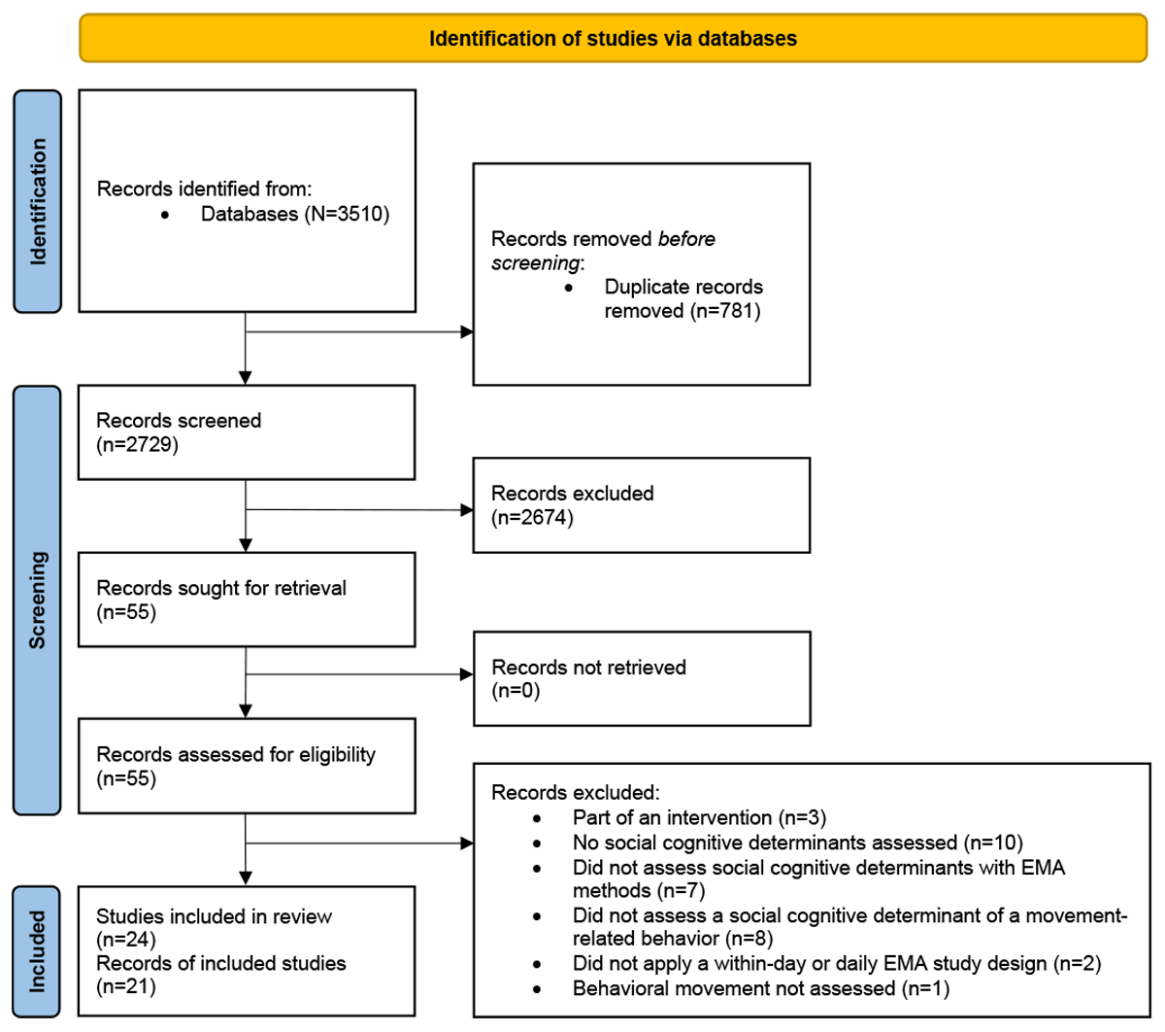
PRISMA (Preferred Reporting Items for Systematic Reviews and Meta-Analyses) flow diagram for the literature search on social cognitive determinants, movement-related behaviors, and ecological momentary assessment (EMA) methods.

Literature searches were conducted on May 25, 2022, in the PubMed, SPORTDiscus, and PsycINFO databases to identify relevant articles that used EMA methods to examine associations between social cognitive determinants and movement-related behaviors. No date restrictions were applied in the searches. This process is shown in the PRISMA flow diagram in Figure 1. In total, 3 sets of search terms were used to identify potentially relevant articles. The first set of search terms used general terms, including the following: (“physical activity” OR “exercise” OR “sedentary behavior” OR “movement behavior” OR “physical exercise” OR “sitting”) AND (“ecological momentary assessment” OR “EMA” OR “daily diary” OR “experience sampling”). The second set of search terms included the general terms (from the first search) along with specific terms related to psychological determinants: (“social cognitive” OR “motivation” OR “psychosocial” OR “behavioral cognitions”). The third set of search terms included the general terms from the first search along with specific terms related to social cognitive determinants: (“self-efficacy” OR “outcome expectation” OR “intention” OR “attitude” OR “subjective norm” OR “control” OR “risk perception” OR “barriers” OR “facilitators” OR “goal” OR “plan”). See Multimedia Appendix 1 for the complete search term queries in each database. These specific social cognitive terms were selected by identifying constructs outlined within the Theory of Planned Behavior and Social Cognitive Theory, 2 of the most prominent social cognitive frameworks [6]. The first search using general terms was completed so as to not miss any EMA movement-related behavior articles that may have assessed social cognitive determinants but did not have them as their focus. All articles were collected in Zotero (Corporation for Digital Scholarship) and uploaded to Rayyan (Rayyan Systems Inc), a web tool used to provide support for systematic reviews [40].

### Article Screening and Coding

A total of 9 reviewers were involved in the process of screening and extracting information from the eligible articles. All reviewers received training on EMA studies of movement-​related behaviors and social cognitive determinants from an expert in this content area (JPM). Following training, all reviewers screened the same 50 abstracts to determine whether the full text should be examined for eligibility. All reviewers then met with the content area expert to discuss and resolve discrepancies. Following this, reviewers independently screened assigned abstracts. One reviewer (KMB) screened all abstracts, and one additional reviewer (KYO, RMR, LB, CM, JY, JMS, BLH, or DJH) was assigned to each abstract. Following screening of all abstracts, discrepancies were discussed and resolved between each pair of reviewers with the content area expert.

The articles retained after title and abstract screening were gathered as full texts. In total, 2 reviewers (KMB and JPM) reviewed the full articles independently to determine eligibility and came together to discuss and resolve any discrepancies. A flowchart using the PRISMA 2020 guidelines shows the screening process for eligible articles (Figure 1) [41].

After identifying the articles to be included in this systematic review, 4 reviewers (KMB, RMR, KYO, and BLH) independently extracted relevant information from 8 articles, met with the content area expert (JPM) to discuss and resolve any discrepancies, and then independently coded the remaining articles. One reviewer (KMB) extracted relevant information from all the articles, and one additional reviewer (RMR, KYO, or BLH) was assigned to each article. Finally, the reviewers and content area expert met to discuss and resolve any discrepancies regarding article information extraction.

For each eligible article, the extracted information focused on study participant characteristics, study design characteristics, study main findings, and methodological quality assessments according to 2 established instruments. First, the Methodological Quality Questionnaire (MQQ) [42] assessed overall study quality based on 9 dimensions (ie, theoretical or conceptual definition, research design, sampling design, sample, evidence of reliability and validity, data analysis, implications for practice, and implications for policy). MQQ scores can range from 0 to 27. Second, the Checklist for Reporting Ecological Momentary Assessment Studies (CREMAS) [43] assessed quality with regard to reporting EMA methodology on 5 dimensions that outline specific criteria that have to be included in the title, introduction, methods, results, and discussion. Across these 5 dimensions, there were 16 items (ie, title and keywords, rationale, training, technology, wave duration, monitoring period, prompting design, prompt frequency, design features, attrition, prompt delivery, latency, compliance rate, missing data, limitations, and conclusions).

CREMAS scores can range from 0 to 16, with 1 point per item addressed. For both the MQQ and CREMAS, higher scores indicate better methodological quality.

### Analysis

The associations between social cognitive determinants and movement-related behaviors were assessed using the guidelines developed by Sallis et al [44]. An association was supported if 60% to 100% of the articles reported such an association. No association was supported if 0% to 33% of the articles reported an association. An indeterminate or inconclusive association was supported if 34% to 59% of the articles reported an association. Statistical significance (*P*<.05) and parameter estimates (and 95% CIs, if reported) were used to determine whether any association between a social cognitive determinant and movement-related behaviors existed and the direction of the association (ie, positive or negative), respectively. All findings from individual articles were presented; however, at least 4 articles were needed to make an assessment regarding an overall association between a given social cognitive determinant and movement-related behavior in this systematic review. The choice to conduct a systematic review of results was based on the substantial diversity of study designs, operationalizations of social cognitive determinants and movement-related behaviors, and analyses to test associations (eg, multilevel linear regression, multilevel logistic regression, multilevel negative binomial model, and time-varying effect modeling).

## Results

### Overview

A total of 3510 articles were identified across all the database searches. After duplicates were removed (781/3510, 22.25%), the remaining articles were screened by title and abstract (2729/3510, 77.75%). After screening, 2.02% (55/2729) of the articles were identified for full-text retrieval. Of those 55 articles, 31 (56%) were excluded, leaving 24 (44%) articles from 21 unique studies to be included in this systematic review (Figure 1). The publication year, sample and study characteristics, and methodological quality scores of each article are presented in Table 1.

**Table 1:** Summary of article characteristics (N=24).

Study	Sample size, N	Age (years), mean (SD)	Sex or gender (% of female and women participants)	Race and ethnicity (% of White or non-Hispanic White participants)	Population	EMA^a^ delivery medium	EMA protocol design	Social cognitive determinants of interest	Behavioral constructs of interest	Behavioral assessment method	CREMAS^b^ rating	MQQ^c^ rating
Arigo et al [45]	75	51.61 (5.43)	100	73	Adult women	Website	5 prompts per day for 10 days; single wave; signal contingent	Intentions	PA^d^	Accelerometer	10	21
Cook et al [46]	55	20-69^e^	15	58	Adults with HIV	Website	1 prompt per day for 30 days; single wave; signal contingent	Self-efficacy	PA	Accelerometer	9	16
Maher et al^f^ [47]	116	40. 3 (9.6)	74.2	46.6	Adults	Smartphone	8 prompts per day for 4 days; multiple waves; signal contingent	Intentions, self-efficacy, and outcome expectations	PA	Accelerometer	15	22
Maher et al^f^ [48]	116	40.3 (9.6)	74.2	46.6	Adults	Smartphone	8 prompts per day for 4 days; multiple waves; signal contingent	Intentions	PA	Accelerometer	15	27
Pickering et al^f^ [49]	116	40.3 (9.6)	74.2	33	Adults	Smartphone	8 prompts per day for 4 days; multiple waves; signal contingent	Intentions, self-efficacy, and outcome expectations	PA	Accelerometer	13	25
Maher and Dunton^g^ [50]	103	72 (7)	62	74	Older adults	Smartphone	6 prompts per day for 10 days; single wave; signal contingent	Intentions and self-efficacy	SB^h^	Accelerometer	15	27
Maher and Dunton^g^ [51]	103	72.4 (7.44)	62.5	67.3	Older adults	Smartphone	6 prompts per day for 10 days; single wave; signal contingent	Intentions and self-efficacy	PA and SB	Accelerometer	15	27
Reifsteck et al [52]	17	21.82 (0.64)	47.06	82.35	College student athletes	Smartphone	4 prompts per day for 7 days; single wave; signal contingent	Intentions, self-efficacy, and outcome expectations	PA	Accelerometer	14	18
Zenk et al [53]	97	25-65^e^	100	0	Adult women	Website	5 prompts per day^i^ for 7 days; single wave; signal contingent	Barriers	PA and SB	Accelerometer	10	19
Anderson [54]	76	40.29 (13.69)	57.9	85.5	Adults	Website	1 prompt per day for 14 days; single wave; interval contingent	Planning	PA	Self-report	11	27
Carraro and Gaudreau [55]	97	20.45 (4.61)	68	71	College students	Website	1 prompt per day for 6 days; single wave; interval contingent	Action planning and coping planning	PA	Self-report	13	25
Dunton et al [56]	23	60.65 (8.22)	70	91	Older adults	PDA	4 prompts per day for 14 days; single wave; interval contingent	Self-efficacy	PA	Self-report	14	14
McDonald et al [57]	7	62.71	71.4	Not collected^j^	Older adults	PDA	2 prompts per day for a minimum of 2 months (maximum of 7 months); single wave; interval contingent	Intentions and perceived behavioral control	PA	Accelerometer	11	14
Bermudez et al [58]	111	62.32 (10.85)	14.4	Not collected	Adults with cardiac disease	PDA	1 prompt per day for 21 days; single wave; event contingent	Intentions, perceived behavioral control, subjective norms, and explicit attitudes	PA	Accelerometer	8	16
Berli et al [59]	120	Women: 43.39 (12.67); men: 45.07 (13.92)	50	Not collected	Adult dual-smoker couples	Smartphone	1 prompt per day for 28 days; single wave; event contingent	Intentions, self-efficacy, and action planning	PA	Accelerometer	12	23
Bond et al [60]	21	48.5 (2.8)	81	71.4	Adults who had undergone bariatric surgery	PDA	1 prompt per day for 6 days; single wave; event contingent	Intentions	PA	Self-report	11	24
Borowski et al [61]	61	41.4 (9.9)	88.5	90.2	Adults	Website	1 prompt per day for 7 days; single wave; event contingent	Barriers	PA	Self-report	10	21
Conroy et al [62]	63	Not collected	58.7	87	College students	Website	1 prompt per day for 14 days; single wave; event contingent	Intentions	PA	Self-report	14	23
Conroy et al [63]	128	21.3 (1.1)	58.6	89	College students	Website	1 prompt per day for 14 days; single wave; event contingent	Intentions	SB	Accelerometer	12	23
Curtis et al [64]	185	61.7 (5.9)	55.1	Not collected	Older adults	Website	1 prompt per day for 7 days; single wave; event contingent	Self-efficacy	PA	Self-report	10	18
Maher and Conroy [65]	100	74.2 (8.2)	67	99	Older adults	PDA	2 prompts per day for 14 days; single wave; event contingent	Intentions, self-efficacy, action planning, and coping planning	SB	Accelerometer and self-report	12	23
Rebar et al [66]	103	21.57 (2.97)	52	Not collected	College students	Website	1 prompt per day for 7 days; single wave; event contingent	Intentions	PA	Self-report	11	21
Schwaninger et al [67]	198	Women: 45.31 (13.51); men: 47.29 (13.94)	50	Not collected	Adult couples	Smartphone	1 prompt per day for 14 days; single wave; event contingent	Self-efficacy	PA	Accelerometer	8	15
Zhaoyang et al [68]	135	65.71 (9.83)	56.3	86.67	Older adult couples with knee OA^k^	PDA	2 prompts per day for 22 days; single wave; event contingent	Self-efficacy	PA	Accelerometer	13	25

^a^EMA: ecological momentary assessment.

^b^CREMAS: Checklist for Reporting Ecological Momentary Assessment Studies. Lowest score=0; highest score=16.

^c^MQQ: Methodological Quality Questionnaire. Lowest score=0; highest score=27.

^d^PA: physical activity.

^e^Only age range was reported.

^f^Articles used data from the same study.

^g^Articles used data from the same study.

^h^SB: sedentary behavior.

^i^Although the study assessed the constructs of interest within days, the data were aggregated to the day level for analysis.

^j^Participants were not asked about this variable.

^k^OA: osteoarthritis.

### Study Sample Characteristics

The analytic sample size of each study ranged from 7 to 198 participants, with a mean sample size of 90.05 (SD 50.70). All articles except for the one by Reifsteck et al [52] (23/24, 96%) reported on studies with samples in which at least 50% of the participants identified as women. The samples reported in 21% (5/24) of the articles were college or university students [52,55,62,63,66]. The samples reported in 50% (12/24) of the articles focused on adults [45-49,53,54,58-61,67]. The samples reported in 29% (7/24) of the articles were older adults (aged ≥50 years) [50,51,56,57,64,65,68]. The mean age of the participants from the 21 unique studies was 47.65 (SD 17.84) years. None of the studies included samples of children or adolescents. Of the 18 articles that reported on participants’ racial or ethnic identities, 4 (22%; n=3, 75% using data from the same study) [47-49] indicated that participants identifying as White or non-Hispanic White did not make up most of the sample [47-49,53].

### EMA Protocol

Of the 24 articles, 21 (88%) reported on studies that collected data over 1 wave. A total of 12% (3/24) of the articles (using data from the same study) reported that data were collected across 3 measurement waves spaced over 1 year [47-49]. Regardless of the number of waves, the monitoring period in each study ranged from 4 to 30 days, except for 4% (1/24) of the articles, which sampled participants daily for 2 to 7 months [57]. Regarding EMA prompting design, of the 24 articles, 12 (50%) reported on studies that had 1 prompt per day [46,54,55,58-64,66,67], 3 (12%) reported on studies that had 2 prompts per day (ie, one in the morning and one in the evening) [57,65,68], and the remaining articles (9/24, 38%) reported on studies that delivered between 4 and 8 prompts per day [45,47-53,56].

In total, 38% (9/24) of the articles indicated that EMA prompting was a signal-contingent design (ie, occurring at randomly prompted times) [45-53]. A total of 17% (4/24) of the articles reported on studies that used interval-contingent design (ie, occurring at fixed times) [52,54,56,57], whereas 46% (11/24) of the articles reported on studies that used event-contingent design (eg, self-initiated EMA questionnaire after a specific event) [55,58-65,67,68].

Regarding design considerations to reduce participant burden, the studies reported in 17% (4/24) of the articles did not ask about social cognitive determinants at every EMA prompt to limit the number of items in each prompt [47-49,56]. The studies reported in 8% (2/24) of the articles customized the time of the prompts based on each participant’s wake and sleep schedules [45,57], whereas another study had participants select their own start day to fit their work schedule [61].

EMA protocols were delivered via smartphone (8/24, 33%), PDA (6/24, 25%), or website (10/24, 42%). Among the articles that reported on studies that used a smartphone to deliver EMA protocols, commercially available apps including Personal Analytics Companion (1/24, 4%) [66], movisensXS (2/24, 8%) [50,51], and MyExperience (3/24, 12%) [47-49] were used. A total of 8% (2/24) of the articles reported on studies that had participants complete the study protocol on a smartphone but did not specify the platform used to deliver the EMA prompts [59,67]. PDA devices comprised handheld computers (3/24, 12%) [56,60,68], tablets (2/24, 8%) [58,65], and a wrist-worn Patient-Reported Outcomes Diary device (1/24, 4%) [57]. Articles on studies that used websites either did not specify the website [45,52,53,55,62-64] or did specify it using Prolific [54], Qualtrics [61], or REDCap (Research Electronic Data Capture; Vanderbilt University) [46].

All articles (24/24, 100%) reported participants’ compliance or response rates within the EMA protocol. These rates ranged from 56.9% to 95%, indicating general compliance with the EMA methodology.

### Social Cognitive Determinants

Intentions (15/24, 62%) [45,47-51,55,57-60,62,63,65,66] and self-efficacy (14/24, 58%) [46,47,49-51,56-59,64-68] were the most frequently assessed social cognitive determinants. Of the 14 articles categorized as reporting on studies that assessed self-efficacy [57,58], 2 (14%) operationalized their construct of interest as perceived behavioral control; however, perceived behavioral control is generally considered synonymous with self-efficacy [9,69], so the findings were combined into 1 category for this review. Other social cognitive determinants assessed included planning (4/24, 17%) [52,54,59,65], outcome expectations (3/24, 12%) [47,49,66], barriers (2/24, 8%) [53,61], and explicit attitudes (1/24, 4%) [58].

Intentions and self-efficacy were primarily assessed using 1 to 2 items, but 4% (1/24) of the articles reported on studies that used 4 items to assess self-efficacy [46]. The studies reported in 29% (7/24) of the articles adapted social cognitive determinant items from validated scales [46,54,55,58,59,61,65], but almost all (5/7, 71%) reduced the number of items or adapted the time frame of the items for delivery as part of their EMA protocol. Common behavioral targets assessed with social cognitive determinant items included engaging in PA [46,53-56,58-62,64,66-68], engaging in a specified duration of PA [45,47-52,57], or limiting SB to a specified total amount of time [53,63,65,66].

### Movement-Related Behaviors

Methods for assessing movement-related behaviors included device-based (15/24, 62%) and self-reported (8/24, 33%) assessments or both (1/24, 4%) [65]. Common devices used included the ActiGraph GT3x or GT3x+ (7/24, 29%) [45,55,58,59,63,66,67], ActiGraph GT2M (3/24, 12%) [47-49], ActiGraph GT1M (1/24, 4%) [53], Fitbit Alta HR (1/24, 4%) [46], and activPAL 3 (3/24, 12%) [50,51,65]. A total of 4% (1/24) of the articles reported on studies that provided participants with either the ActiGraph GT1M or GT3x+ device [68], and 4% (1/24) used the Patient-Reported Outcomes Diary device [57]. In total, 75% (18/24) of the articles reported on studies that required a minimum threshold of valid wear time for data to be included in the analysis [45-53,56,58,​^59^,^61^,^63^,^65^-^68^]. Minutes of moderate- to vigorous-intensity PA (MVPA) were the most common operationalization of PA (12/24, 50%) [45,47-49,53,55,56,58,59,63,66,67], and minutes of sedentary time were the most common operationalization of SB (5/24, 21%) [50,51,53,55,65] using device-based measures.

Among the self-reported measures of movement-related behaviors, the studies reported in 25% (6/24) of the articles adapted items from existing validated measures, including the International Physical Activity Questionnaire–Short Form by Sjöström et al [63,65], Godin Leisure-Time Exercise Questionnaire by Godin and Shephard [52,54,61], and a measure of sedentary time in older adults developed by Gardiner et al [65]. Other assessments of daily movement-related behaviors included checklists in which participants indicated the activities they had participated in that day and for how long [56,64]. Bond et al [60] and Rebar et al [66] assessed the daily duration of MVPA using items created for the study.

### Methodological Quality

The average MQQ score was 21.38 (SD 4.31; range 14-27), with 21% (5/24) of the articles scoring 27 (the highest possible score). The interrater reliability for the MQQ scores was 98.3%, which is an acceptable level of agreement [55]. On the basis of the 9 criteria of the MQQ, the most frequently omitted information pertained to evidence of reliability and validity provided for the data collected (12/24, 50%) [45,46,53,56-58,60-63,66,67] followed by implications for policy (11/24, 46%) [45,46,53,55-59,61,64,67].

The average CREMAS score was 11.92 (SD 2.21; range 8-15). The interrater reliability for the CREMAS scores was 93.75%. Commonly omitted elements of the CREMAS among applicable articles included participant training procedures, latency, and missing data analysis. Of the 20 articles in which latency was applicable (eg, interval- and signal-contingent designs), none reported latency. A total of 54% (13/24) of the articles did not report any missing data analyses [45,46,52-54,58-61,64-67]. In total, 50% (12/24) of the articles did not report any training to familiarize participants with the EMA protocol [46,49,53-56,58,61,64,66-68]. Except for the studies reported in 12% (3/24) of the articles [47-49], wave duration (ie, the number of data collection waves in a study) was not applicable as all other studies collected data over a single wave.

### PA and Social Cognitive Determinants

This systematic review identified studies examining associations between specific social cognitive determinants (ie, intentions, self-efficacy, outcome expectations, planning, perceived barriers, and attitudes) and PA; however, the availability of data regarding these associations differed at the momentary and daily levels. The findings are summarized in the following sections.

#### Intentions

##### Momentary Associations

The studies reported in 25% (6/24) of the articles assessed associations between intentions and PA at the momentary level, which is shown in Table 2. Of those 6 articles, 3 (50%) focused on direct relationships between momentary intentions and subsequent PA. Among college student athletes, Reifsteck et al [52] documented a positive association between intentions and behavior such that, on occasions when participants reported stronger-than-usual intentions, they engaged in more device-based MVPA over the following 3 hours. Similarly, Arigo et al [45] found a weak positive association between the number of intended minutes and minutes of device-based MVPA over the following 3 hours among adults, although the results were not significant. However, this positive association between intentions and behavior became stronger and more significant on occasions when participants experienced less contentment or body satisfaction than usual. Conversely, Pickering et al [49] found a null association between momentary intentions and subsequent device-based MVPA among adults. However, on occasions when adults had higher self-efficacy to engage in PA than was typical for them, momentary intentions were positively associated with subsequent MVPA. As only 12% (3/24) of the articles reported on studies that investigated momentary intention–PA relationships and we determined a priori that there must be at least 4 articles present to draw a conclusion regarding an overall association, this cannot be made at this time.

In total, 12% (3/24) of the articles focused specifically on time-varying moderators of intention-PA relationships, with all articles (3/3, 100%) reporting on studies that used device-based measures of PA. Time of day and day of the week were moderators investigated by Maher et al [47,51]. Time-varying effect models applied by Maher et al [47] revealed that intentions to be physically active positively predicted subsequent MVPA in the mornings and evenings but not in the afternoons. On weekdays, intentions were unrelated to subsequent PA on weekends. Using a similar approach, Maher and Dunton [51] found that, among older adults, intentions to be active were positively associated with subsequent time spent upright (ie, standing or stepping) during the morning, afternoon, and evening on both weekdays and weekends, although the magnitude of the associations changed throughout the day. Finally, Maher et al [48] investigated affect states and physical context as moderators of the intention-PA coupling and found that individuals were more likely to follow through with their intentions to be physically active on occasions when they reported greater positive affect than was typical for them (at the same time that they reported their intentions). Owing to the range of time-varying moderators investigated, conclusions regarding consistent moderators of intention-PA relationships at the momentary level cannot be drawn.

**Table 2:** Associations between social cognitive determinants and physical activity and sedentary behavior.^a^

Social cognitive determinant and timescale of associations			Positive association	Negative association	Null association	Moderators	Overall association
**Physical activity**							
	**Intentions predicting physical activity**						
		Momentary associations	Reifsteck et al [52] Arigo et al [45]	No articles reported a negative association	Pickering et al [49]	Self-efficacy: Pickering et al [49] Time of day: Maher et al [47] and Maher and Dunton [51] Day of the week: Maher et al [47] and Maher and Dunton [51] Positive affect: Maher et al [48]	N/A^b^
		Daily associations	Conroy et al [62] Berli et al [59] Bermudez et al [58] Bond et al [60] McDonald et al [57]	McDonald et al [57]	Rebar et al [66] McDonald et al [57]	Ego depletion: Rebar et al [66]	+^c^
	**Self-efficacy predicting physical activity**						
		Momentary associations	Cook et al [46] Dunton et al [56]	No articles reported a negative association	Reifsteck et al [52] Cook et al [46] Pickering et al [49]	Intentions: Pickering et al [49] Time of day: Maher et al [47] and Maher and Dunton [51] Day of the week: Maher et al [47] and Maher and Dunton [51]	?^d^
		Daily associations	Berli et al [59] Schwaninger et al [67] Curtis et al [64] McDonald et al [57] Zhaoyang et al [68]	Bermudez et al [58] McDonald et al [57]	Bermudez et al [58] McDonald et al [57]	Age: Curtis et al [64]	+
	**Outcome expectations predicting physical activity**						
		Momentary associations	Reifsteck et al [52]	No articles reported a negative association	Maher et al [47] Pickering et al [49]	No articles assessed moderators	N/A
	**Planning predicting physical activity**						
		Daily associations	Carraro and Gaudreau [55] Anderson [54] Berli et al [59]	No articles reported a negative association	Carraro and Gaudreau [55]	Typical plans: Anderson [54] Goal conflict: Carraro and Gaudreau [55]	N/A
	**Barriers predicting physical activity**						
		Daily associations	No articles reported a positive association	Borowski et al [61] Zenk et al [53]	Zenk et al [53]	No articles assessed moderators	N/A
	**Attitudes predicting physical activity**						
		Daily associations	No articles reported a positive association	No articles reported a negative association	Bermudez et al [58]	No articles assessed moderators	N/A
**Sedentary behavior**							
	**Intentions predicting sedentary behavior**						
		Momentary associations	No articles reported a positive association	Maher and Dunton [50]	No articles reported a null association	Time of day: Maher and Dunton [51] Day of the week: Maher and Dunton [51]	N/A
		Daily associations	No articles reported a positive association	Conroy et al [63]	No articles reported a null association	No articles assessed moderators	N/A
	**Self-efficacy predicting sedentary behavior**						
		Momentary associations	No articles reported a positive association	Maher and Dunton [50]	No articles reported a null association	Time of day: Maher and Dunton [51] Day of the week: Maher and Dunton [51]	N/A
	**Planning predicting sedentary behavior**						
		Daily associations	No articles reported a positive association	Maher and Conroy [65]	No articles reported a null association	No articles assessed moderators	N/A
	**Barriers predicting sedentary behavior**						
		Daily associations	No articles reported a positive association	No articles reported a negative association	Zenk et al [53]	No articles assessed moderators	N/A

^a^A total of 4 articles were needed to make an overall association.

^b^N/A: not applicable; <4 articles on the social cognitive determinant, so an overall association cannot be determined.

^c^+: Positive association (≥60% of the studies showing an association).

^d^?: inconclusive (34%-59% of the studies showing an association).

##### Daily Associations

The studies reported in 25% (6/24) of the articles assessed the association between intentions and PA at the day level. Of these 6 articles, 4 (67%) documented positive associations between daily intentions and behavior regardless of whether intentions were assessed upon waking [60] or the previous evening [59]. Furthermore, these associations were consistent across different operationalizations of PA, including self-reported MVPA [60,62] and device-based MVPA [57-59]. Conversely, Rebar et al [66] found a null association between intentions to exercise the following day and self-reported exercise; however, on nights when university students experienced more ego depletion (limited cognitive and physical capabilities), they were more likely to successfully enact those exercise intentions the following day. McDonald et al [57] observed older adults in the months leading up to and following retirement and found that intentions did not consistently predict PA in all participants. The findings indicate that intentions to be active were positively associated with (1/24, 4%), negatively associated with (1/24, 4%), or not related to (5/24, 21%) likelihood of engaging in a PA bout depending on the participant. On the basis of the criteria by Sallis et al [44], the findings indicate an overall positive association between intentions and subsequent PA at the day level (ie, ≥60 of the articles; 4/6, 67% reported a consistent positive association); however, further investigation of moderators of these daily associations is warranted.

#### Self-efficacy

##### Momentary Associations

The studies reported in 25% (6/24) of the articles assessed associations between self-efficacy and PA at the momentary level, which is shown in Table 2. Of these 6 articles, 4 (67%) focused on direct relationships between momentary self-efficacy and subsequent PA. Among older adults, Dunton et al [56] found a positive association between momentary self-efficacy and subsequent self-reported MVPA. Similarly, Cook et al [46] found a positive association between momentary self-efficacy and device-based MVPA, although a null association was found between momentary self-efficacy and total steps.

Conversely, both Pickering et al [49] and Reifsteck et al [52] found null associations between momentary self-efficacy and subsequent device-based MVPA among adults and college student athletes, respectively. Pickering et al [49] did find that momentary intentions moderated momentary self-efficacy–​behavior relationships such that, on occasions when adults had stronger intentions to engage in PA than usual, momentary self-efficacy was positively associated with subsequent MVPA. On the basis of the criteria by Sallis et al [44] and because of mixed findings across these articles, an inconclusive overall association was found between momentary self-efficacy and PA (ie, 12/24, 50% of the articles reported a consistent positive association).

The studies reported in 8% (2/24) of the articles specifically explored moderators of self-efficacy–PA relationships at the momentary level. Maher et al [47,51] investigated differences in self-efficacy and PA associations by time of day and day of the week. Among adults, self-efficacy was positively associated with device-based MVPA on weekday evenings [47]. At no other time on weekdays or weekends was self-efficacy associated with subsequent PA. Among older adults, self-efficacy was positively associated with device-based time spent upright (standing or stepping) all day on weekdays and during the mornings and afternoons but not in the evenings on weekends [51]. These findings point to potential temporal moderators of self-efficacy–PA relationships at the momentary level; however, as only 8% (2/24) of the articles reported on studies that investigated these temporal processes, firm conclusions cannot be drawn.

##### Daily Associations

The studies reported in 25% (6/24) of the articles examined associations between self-efficacy and PA at the day level. Of these 6 articles, 4 (67%) documented positive associations between self-efficacy and subsequent PA on a given day. Findings were consistent across self-reported [64] and device-based [59,67,68] MVPA as well as ratings of self-efficacy that occurred retrospectively (ie, self-efficacy over the previous 24 hours [64], the previous evening [59,67], and upon waking [68]). In addition, Curtis et al [64] did document moderation by age such that, for older adults aged >70 years, daily self-efficacy was positively associated with higher levels of self-reported PA, whereas daily self-efficacy was not associated with PA for participants aged 51 to 69 years. Throughout the retirement transition, McDonald et al [57] found that, for 2 participants (out of 7), stronger feelings of perceived behavioral control (which is argued to be synonymous with self-efficacy [9]) were associated with a greater likelihood of engaging in a device-captured PA bout on the same day. However, 1 participant had a negative association, and 4 participants had no association between these constructs. Contrary to the relationships hypothesized within social cognitive frameworks, Bermudez et al [58] documented a null association among perceived behavioral control, device-based MVPA, and light-intensity PA at the day level. Furthermore, the same study documented a negative association between usual levels of perceived behavioral control and light-intensity PA, where higher usual levels (as opposed to on a given day) of perceived behavioral control were associated with lower levels of light-intensity PA. On the basis of the criteria by Sallis et al [44], the findings indicate a positive association between self-efficacy and PA at the day level (ie, ≥60% of the articles—4/6, 67%—reported consistent positive associations); however, further investigation of moderators of these daily associations is necessary.

#### Outcome Expectations

##### Momentary Associations

The studies reported in 12% (3/24) of the articles assessed outcome expectations and PA at the momentary level, shown in Table 2. Among college student athletes, Reifsteck et al [52] found that, on occasions when outcome expectations regarding PA were higher than usual, individuals engaged in more device-based MVPA over the following 3 hours. However, using an identical measure of outcome expectations and PA as in the study by Reifsteck et al [52], Pickering et al [49] found null associations between momentary outcome expectations and PA among adults. Furthermore, Maher et al [47] found that momentary outcome expectations were not associated with subsequent PA regardless of time of day or day of the week. As only 12% (3/24) of the articles reported on studies that investigated momentary outcome expectation–PA relationships and we determined a priori that there must be at least 4 articles present to draw a conclusion regarding an overall association, this cannot be made at this time.

##### Daily Associations

None of the articles reported on studies that examined associations between daily outcome expectations and PA.

#### Planning

##### Momentary Associations

None of the articles reported on studies that examined associations between planning and PA at the momentary level.

##### Daily Associations

The studies reported in 12% (3/24) of the articles examined associations between daily planning and PA. Of these 3 articles, 1 (33%) reported on a study that investigated general planning [54], 1 (33%) reported on a study that investigated action planning [59], and 1 (33%) focused on action and coping planning [55]. General planning and action planning were both found to be positively associated with self-reported [54,55] and device-based [59] daily MVPA; however, daily coping planning and self-reported PA were not associated [55]. As only 12% (3/24) of the articles reported on studies that investigated daily planning–PA relationships and we determined a priori that there must be at least 4 articles present to draw a conclusion regarding an overall association, this cannot be made at this time.

Regarding moderators, Anderson [54] found that usual levels of planning moderated associations between daily planning and PA such that, for individuals who tended to have weaker PA planning, on days when they reported stronger-than-usual plans to be active, they engaged in more self-reported MVPA. Carraro and Gaudreau [55] found that a time-varying factor—daily academic goal conflict—moderated associations between daily action planning and PA such that daily action planning was positively associated with daily self-reported PA on days during which individuals experienced lower academic goal conflict. Although these studies suggest possible time-invariant and time-varying moderators of daily planning–PA relationships, because of the limited number of studies investigating such moderators, conclusions cannot be drawn at this time.

#### Perceived Barriers

##### Momentary Associations

None of the articles reported on studies that examined associations between momentary barriers and PA.

##### Daily Associations

Borowski et al [61] documented a negative association between daily barriers to exercise (eg, no time, feeling tired, and air or noise pollution) and self-reported PA, and for each additional barrier reported, participants engaged in 27% fewer minutes of PA that day. Zenk et al [53] examined associations between different types of barriers and device-based PA and found that the extent to which African American women endorsed poor weather as a barrier to PA was associated with less PA but that environmental (eg, no sidewalk or no indoor facilities) and social (eg, no one to exercise with and safety or crime concerns) barriers were not associated with PA. Owing to the limited number of articles, an overall association between daily barriers and PA cannot be determined at this time.

#### Attitudes

##### Momentary Associations

None of the articles reported on studies that examined associations between momentary attitudes and PA.

##### Daily Associations

Bermudez et al [58] found that neither affective nor instrumental attitudes on a given day were associated with device-based MVPA or light-intensity PA. As only 4% (1/24) of the studies investigated this topic, an overall association between attitudes and PA cannot be determined at this time.

### SB and Social Cognitive Determinants

#### Overview

This systematic review identified studies examining associations between specific social cognitive determinants (ie, intentions, self-efficacy, planning, and perceived barriers) and SB, but the availability of data regarding these associations differed at the momentary and daily levels. However, because of the limited number of studies investigating such associations, overall associations between each social cognitive determinant and SB cannot be determined at this time. Nevertheless, we report our findings in the following sections.

#### Intentions

##### Momentary Associations

Maher and Dunton [50] found that, on occasions when older adults had stronger intentions than usual to limit their SB, they subsequently engaged in less device-based SB in the following 2-hour period. Using the same data set, Maher and Dunton [51] found that, on weekdays, momentary intentions to limit SB negatively predicted subsequent SB across the entire day, but on weekends, intentions only negatively predicted SB in the morning, afternoon, and early evening.

##### Daily Associations

Conroy et al [63] examined day-level associations between end-of-day intentions to limit SB and next-day SB among university students and found that, on days when university students had stronger-than-usual intentions to limit their SB, they subsequently engaged in less self-reported SB the following day.

#### Self-efficacy

##### Momentary Associations

Maher et al [50,51] have published 2 articles examining associations between momentary self-efficacy and SB. Maher and Dunton [50] found that, on occasions when older adults had stronger self-efficacy to limit SB, they subsequently engaged in less device-based SB over the following 2 hours. Investigation of day of the week and time of day as moderators of this association revealed that self-efficacy to limit SB was negatively associated with SB throughout the day on weekdays but that, on weekends, self-efficacy to limit SB was only associated with subsequent SB in the afternoon [51].

##### Daily Associations

None of the articles reported on studies that examined associations between daily self-efficacy and SB.

#### Planning

##### Momentary Associations

None of the articles reported on studies that examined associations between momentary plans and SB.

##### Daily Associations

Maher and Conroy [65] investigated daily associations between planning (ie, a composite score of action and coping planning) and SB and found that, on mornings when plans were stronger than usual to limit SB, older adults engaged in less device-based SB that day.

#### Perceived Barriers

##### Momentary Associations

None of the articles reported on studies that examined associations between barriers and SB.

##### Daily Associations

Zenk et al [53] found null associations between weather, environment, and social barriers to engaging in PA and device-based SB on a given day among African American women.

## Discussion

### Principal Findings

This review is the first to summarize the available EMA evidence of associations between social cognitive determinants and movement-related behaviors at the momentary and daily levels. Although this review included 24 articles comprising 21 unique studies, there were limited studies investigating each individual social cognitive determinant’s relationship with subsequent behavior, especially after accounting for the timescale of assessment (ie, momentary level vs day level). The largest evidence base and, therefore, the strongest conclusions in the systematic review pertain to relationships between intentions and PA and between self-efficacy and PA. Overall, synthesizing the available evidence contributes to our preliminary understanding of the impact of social cognitive determinants on subsequent movement-related behaviors in real-world environments, identifies gaps in the literature to direct future movement-related behavior EMA research, and can begin to inform intervention efforts that are designed to deliver contextually relevant motivation content during periods of opportunity and vulnerability.

This systematic review suggests that positive associations exist among intentions, self-efficacy, and PA at the day level. It is not surprising that intentions and self-efficacy, which are prominent constructs posited to directly influence behavior within social cognitive frameworks [70,71], emerged as consistent and positive predictors of PA at the day level in this systematic review. However, articles that reported on studies investigating associations among intentions, self-efficacy, and PA at the momentary level (6/24, 25% and 6/24, 25%, respectively) revealed mixed findings. For instance, although a sufficient number of studies investigated associations between momentary self-efficacy and subsequent PA to determine an overall association, conflicting findings across the studies resulted in an inconclusive overall association. Several time-varying moderators were documented across the articles at the momentary level. It is possible that associations between momentary social cognitive determinants and subsequent PA may be affected by contextual factors that change across time and space as individuals navigate their daily lives, whereas day-level associations may be less affected by immediate contextual features of one’s current environment.

This systematic review suggests that more research is needed to better understand associations between social cognitive determinants and subsequent movement-related behaviors in the context of everyday life. For instance, results from this systematic review suggest that no studies used daily or within-day EMA methodology to examine relationships between PA facilitators (eg, optimal weather) and subsequent PA behavior or between outcome expectations and attitudes and subsequent SB. In addition, there were not enough eligible articles that reported on studies investigating relationships between any social cognitive determinant and SB to draw conclusions. In addition, although some social cognitive determinants such as daily planning appeared to indicate consistent associations with PA across the studies, <4 studies investigated this association, which prevented a conclusion from being drawn. Further complicating the state of the literature in this area is that a variety of measures were used to assess both social cognitive determinants and behavior. Such diversity may have contributed to the discrepant findings across the studies. For instance, among the studies investigating associations between momentary self-efficacy and PA (where an inconclusive overall association was determined; only 2/4, 50% indicated a positive association), Dunton et al [56] assessed participants’ confidence in engaging in PA and found a positive association with PA, whereas Pickering et al [49] assessed participants’ confidence in engaging in PA despite possible barriers and found a null association. However, even across the diverse assessments used, we were able to find consistent trends in relationships between some social cognitive determinants and behaviors (eg, daily associations between intentions and PA), increasing the confidence in our conclusions on those relationships as the diversity of measures reduces the likelihood that the effect is the result of measurement bias. Greater consistency across the studies in the assessment of social cognitive determinants and behavior would allow for more precise conclusions on associations between social cognitive determinants and movement-related behavior and would be more appropriate to quantitatively estimate associations in a meta-analysis.

### Limitations of This Review

Although studies using EMA methods can uncover more nuanced associations between social cognitive determinants and movement-related behaviors by collecting ecologically valid and intensive longitudinal data, the limitations of the studies included in this review should be noted. First, the findings synthesized in this review may not apply to all developmental periods across the life span or individuals of racially or ethnically minoritized backgrounds. No studies included in this review focused on child or adolescent samples, and almost all studies (12/14, 86% that reported race and ethnicity) featured samples in which most participants identified as White or non-Hispanic White. Given that PA levels decline rapidly throughout late childhood and adolescence and racial and ethnic minorities experience physical inactivity–related health disparities [72], EMA may be a critical methodological tool to understand relationships between motivation and behavior in these vulnerable populations. In addition, the studies featured in this review comprised insufficiently active or sedentary individuals as well as those who were sufficiently active; therefore, it is likely that the results are aggregated across individuals at various stages of the behavior change continuum. There is evidence suggesting that social cognitive determinants may differentially regulate behavior across the behavior change continuum, which is an important direction to explore in EMA work to understand movement-related behaviors [24,73]. Further influencing the generalizability of the findings is the lack of missing data analysis in several studies included in this review (12/24, 50%). Such an analysis is essential in EMA studies to determine if data are missing at random or in systematic patterns, which may influence the extent to which documented associations translate to all occasions or all people [43,74].

Most EMA studies included in this review (16/24, 67%) created measures to assess social cognitive determinants or behavior. Although such an approach is common in EMA research as fewer items are used to limit participant burden and fatigue that may result from repeated, intensive assessments [29], the items used in the studies in this review rarely presented psychometric data to establish the validity or reliability of the measures, and the studies often appeared to create items based on face validity. More rigorous and systematic approaches are necessary to develop EMA items to assess social cognitive determinants and movement-related behaviors (for a more in-depth discussion, see the study by Reichert et al [75]).

Furthermore, the limitations of the systematic review itself should be addressed. Although this review assessed the quality of the studies and study reporting through the MQQ and CREMAS, respectively, the information was not used in the interpretation of the results. Neither quality assessment tool specifies thresholds for low-, medium-, or high-quality articles. Therefore, we chose to present the raw scores on each assessment tool. On the basis of those scores, of the 24 articles included in the review, only 5 (21%) scored <18 on the MQQ, and 7 (29%) scored <11 on the CREMAS, which would indicate that those articles satisfied less than two-thirds of the criteria specified within the respective quality assessment tools.

In addition, the inclusion and exclusion criteria of this review did not place any restrictions on the participant sample size necessary for inclusion. A small participant sample size can affect the likelihood of obtaining significant results as well as the magnitude of the association documented. Only 17% (4/24) of the articles included in this review reported on studies that had <50 participants—a threshold determined through simulation to provide adequate power for variance, SE, and fixed-effects estimates at both the between- and within-person levels [76,77]. However, the findings presented in this review primarily focus on within-person findings (or the extent to which social cognitive determinants predict subsequent behavior on a given day or moment). Therefore, power for the analysis is derived from the number of occasions in the analytic sample, and because of the intensive assessments that are a hallmark of EMA, studies typically generate many observations per person. For instance, the study by McDonald et al [57] had the smallest participant sample size of the studies included in the review (N=7) but collected daily assessments from 2 to 7 months, resulting in between 87 and 196 observations per participant. Furthermore, the size of the associations was not considered to fully interpret the relationships documented across the studies as few (7/24, 29%) reported effect sizes. As the volume of studies in this area increases, a meta-analysis should be conducted that accounts for the sample size and effect sizes of relevant studies to further characterize momentary and daily associations between social cognitive determinants and movement-related behaviors.

### Future Directions

PA and SB are considered independent health behaviors with different processes regulating each behavior and different health consequences associated with each [78]; however, most of the research investigating relationships between social cognitive determinants and movement-related behaviors focuses on PA. Given the prevalence of excessive SB as well as recent calls to design movement-related behavior interventions to initially focus on reducing SB and over time build to engaging in MVPA [79,80], EMA studies specifically addressing social cognitive determinant–SB relationships are necessary to develop and refine theoretical frameworks to explain and predict SB.

Across both behaviors, intentions and self-efficacy were the most investigated social cognitive determinants. This is not surprising as the Theory of Planned Behavior and Social Cognitive Theory posit that intentions and self-efficacy are proximal determinants of behavior, respectively [7,9,10,39]. However, based on preliminary evidence from this review, momentary self-efficacy and daily planning may also be important motivational determinants of behavior, but this is based on a limited number of studies. Interestingly, the relationships between planning and PA seemed to depend on the type of planning used (eg, action planning vs coping planning), but it is unclear why these differences might exist across microtimescales given the consistent finding across macrotimescales [81]. Perhaps associations between coping planning and movement-related behavior on microtimescales depend on whether an anticipated barrier is encountered in one’s daily experiences. Similarly, although many studies (12/24, 50%) indicated that self-efficacy was assessed, further inspection of items revealed subtle differences in the type of self-efficacy assessed across the studies (eg, task self-efficacy and barrier self-efficacy), which may be differentially related to behavior and are likely important to consider in future EMA research [82,83].

Findings from this review provide evidence that both time-varying and time-invariant factors can moderate associations between social cognitive determinants and subsequent movement-related behaviors at the momentary and daily levels. A recent systematic review of studies investigating the PA intention-behavior coupling across macrotimescales (eg, weeks and months) found that consistent moderators of the intention-behavior coupling included motivational factors such as intention stability, intention commitment, low goal conflict, affective attitude, anticipated regret, perceived behavioral control or self-efficacy, and exercise identity [69]. Although some EMA studies have investigated some of these motivational factors as moderators [49,55], more work is needed to establish whether between-person moderators across macrotimescales also serve as within-person moderators across microtimescales. In addition, to date, only a handful of studies have investigated the moderating role of affective states and physical and social contexts on social cognitive determinant–behavior relationships [48]. There is recent evidence suggesting that these time-varying contextual factors can influence behavior, and it may be that this influence on behavior is through motivational processes [36,37]. Future research should continue to investigate context-sensitive moderators as this work is an essential first step in theory refinement that is sensitive to the contexts that people encounter in their daily lived experiences [84].

This systematic review focused on observational EMA studies to better understand the naturally occurring relationships between social cognitive determinants and subsequent behavior. However, using intensive assessment methods such as EMA and accelerometry to understand how social cognitive determinant–movement-related behavior relationships change over the course of an intervention may be important for developing more effective behavioral interventions. For instance, Basen-Engquist et al [85] had endometrial cancer survivors complete 3- , 10- , and 12-day EMA protocols spaced over the course of a 6-month PA intervention. Although the authors aggregated data across these 3 time points to determine momentary associations among self-efficacy, outcome expectations, and PA, such data could reveal the extent to which associations between social cognitive determinants and subsequent behavior change over the course of an intervention. Such data could also reveal the extent to which behavior change content delivered at a specific point in the intervention is able to affect social cognitive determinant–behavior relationships. Collecting EMA data regarding social cognitive determinant–behavior relationships may help identify potent intervention content in the context of everyday life.

### Conclusions

This systematic review synthesized EMA-derived associations between social cognitive determinants and subsequent movement-related behavior over microtimescales. Overall, based on the available evidence, social cognitive determinants do regulate movement-related behaviors in the context of everyday life. Specifically, daily intentions and self-efficacy appeared to have a consistent and positive link with PA behavior across the studies using self-reported and device-based measures of behavior. Future research is necessary to determine whether these associations extend to the momentary level, investigate associations among a broader range of social cognitive determinants and movement-related behaviors in diverse populations across the life span, and explore time-varying and time-invariant moderators of these associations. In addition, efforts should be devoted to developing more rigorous study designs, including validating EMA social cognitive determinant assessments and conducting missing data analyses. Ultimately, this systematic review provides foundational knowledge for understanding the motivational determinants of movement-related behaviors within people and is essential for directing future research regarding movement-related behaviors in the context of daily life. In addition, such inquiries are necessary to inform the development of interventions to promote active lifestyles in the context of everyday life.

## Appendix

Multimedia Appendix 1 Full search term queries.

## Abbreviations

CREMAS: Checklist for Reporting Ecological Momentary Assessment Studies
EMA: ecological momentary assessment
MQQ: Methodological Quality Questionnaire
MVPA: moderate- to vigorous-intensity physical activity
PA: physical activity
PRISMA: Preferred Reporting Items for Systematic Reviews and Meta-Analyses
REDCap: Research Electronic Data Capture
SB: sedentary behavior
