# Supplementary material for: Associations Between Social Cognitive Determinants and Movement-Related Behaviors in Studies Using Ecological Momentary Assessment Methods: Systematic Review
Source: JMIR Mhealth Uhealth. 2023 Apr 7;11:e44104. doi: 10.2196/44104 (PMC10131703; doi:10.2196/44104)
Supplement: Multimedia Appendix 1 [file mhealth_v11i1e44104_app1.docx]

**First Set of Search Terms:**

*General Search Terms:*

(‘physical activity’ OR ‘exercise’ OR ‘sedentary behavior’ OR ‘movement behavior’ OR ‘physical exercise’ OR ‘sitting’) AND (‘ecological momentary assessment’ OR ‘EMA’ OR ‘daily dairy’ OR ‘experience sampling’).

**Second Set of Search Terms:**

*General Search Terms and Specific Search Terms Related to Psychological Determinants:*

(‘physical activity’ OR ‘exercise’ OR ‘sedentary behavior’ OR ‘movement behavior’ OR ‘physical exercise’ OR ‘sitting’) AND (‘ecological momentary assessment’ OR ‘EMA’ OR ‘daily dairy’ OR ‘experience sampling’) AND (‘social cognitive’ OR ‘motivation’ OR ‘psychosocial’ OR ‘behavioral cognitions’).

**Third Set of Search Terms:**

*General Search Terms and Specific Terms Related to Social Cognitive Determinants:*

(‘physical activity’ OR ‘exercise’ OR ‘sedentary behavior’ OR ‘movement behavior’ OR ‘physical exercise’ OR ‘sitting’) AND (‘ecological momentary assessment’ OR ‘EMA’ OR ‘daily dairy’ OR ‘experience sampling’) AND (‘self-efficacy’ OR ‘outcome expectation’ OR ‘intention’ OR ‘attitude’ OR ‘subjective norm’ OR ‘control’ OR ‘risk perception’ OR ‘barriers’ OR ‘facilitators’ OR ‘goal’ OR ‘plan’).
